# Tree drawing test as an auxiliary tool for evaluating schizophrenia treatment outcomes

**DOI:** 10.3389/fpsyt.2025.1675521

**Published:** 2025-10-14

**Authors:** Yige Liu, Junmei Yuan, Yanfei Zhang, Hui Jin, Li Gao, Wei Liu, Guorui Liu

**Affiliations:** ^1^ Institute of Applied Psychology, Jiangsu University, Zhenjiang, China; ^2^ Department of Psychiatry, Liaocheng Veterans Hospital, Liaocheng, China; ^3^ Department of Medical Psychology, Second Affiliated Hospital of Naval Medical University, Shanghai, China; ^4^ Psychology Research Institute, Nanchang Vocational University, Nanchang, China; ^5^ School of Digital Media and Design Arts, Beijing University of Posts and Telecommunications, Beijing, China; ^6^ Department of Medical Psychology, No. 905 Hospital of PLA Navy, Shanghai, China

**Keywords:** tree drawing, projective test, schizophrenia, quantitative indicators, treatment evaluation

## Abstract

**Objective:**

This study aimed to investigate the changes in quantitative indicators of the Tree Drawing Projection Test across different treatment stages in patients with schizophrenia, in order to provide an objective basis for evaluating treatment efficacy.

**Methods:**

A self-controlled study design was employed. Sixty patients with schizophrenia underwent the Tree Drawing Projection Test at three time points: Week 1, Week 13, and Week 37. Differences in drawing indicators were analyzed across these time points.

**Results:**

Among patients with schizophrenia, the values of crown area, crown height, crown width, trunk area, and trunk height gradually increased from Week 1 to Week 37, showing observable differences (*p <*0.05). In patients with a disease course of less than 10 years, the changes in trunk area, trunk height, and trunk width during the acute treatment period (Week 1 to Week 13: 5.66 ± 0.55, 2.18 ± 0.20, 0.68 ± 0.08, respectively) were significantly greater than those in patients with a disease course exceeding 10 years (*p <*0.05). At Week 13, schizophrenia patients showed significant differences compared with healthy controls in crown area (99.67 ± 10.89), crown width (11.99 ± 2.15), trunk area (23.94 ± 4.23), trunk width (4.33 ± 0.92), root area (6.43 ± 1.61), and root height (0.76 ± 0.07) (*p <*0.05).

**Conclusion:**

In schizophrenia patients, quantitative indicators of the Tree Drawing Projection Test—specifically crown area, crown height, and crown width—demonstrated steady increases across Week 1, Week 13, and Week 37, with these changes being statistically significant. These findings suggest that the Tree Drawing Projection Test can serve as a useful tool for assessing clinical treatment efficacy in schizophrenia.

## Introduction

Schizophrenia is a common and severe psychiatric disorder with an as-yet unclear etiology. Clinically, it is characterized by disturbances in thought content and affective expression, often accompanied by a disconnection between the individual’s mental activity and the external environment. Cognitive impairments, including deficits in memory, reasoning, and information integration, are also frequently observed (1). Although previous studies have demonstrated a strong genetic component in schizophrenia, no definitive physical or pathological markers currently exist for its diagnosis or for evaluating treatment efficacy. Consequently, the diagnosis primarily relies on detailed patient histories and clinical symptom assessments, typically conducted through mental status examinations based on clinical observation and descriptive psychopathology (2). Currently, treatment outcome evaluations mainly focus on the degree of symptom remission and the recovery of insight (3). Over the years, researchers worldwide have sought more scientific, objective, and effective methods for both diagnosis and therapeutic assessment. In clinical practice, various symptom rating scales are widely used to quantify symptomatology, disease severity, and treatment response. However, these scales are often time-consuming, may impose an additional psychological burden on patients, and carry inherent subjectivity and operational limitations (4).

The Tree Drawing Projection Test, developed by Swiss psychologist Charles Koch in 1952, analyzes the personality and subconscious of individuals by evaluating the characteristics of their drawings. Compared to traditional assessment methods for schizophrenia, the Tree Drawing Test is easier to administer, less time-consuming, and does not require participants to engage in verbal or written expression, thereby reducing psychological pressure. Moreover, its implicit nature helps to conceal the test’s purpose, facilitating the collection of more authentic responses (5, 6). Previous studies have demonstrated that the Tree Drawing Projection Test possesses satisfactory reliability and validity, and some researchers have identified correlations between its indicators and various psychiatric or neurocognitive disorders, such as depressive disorders, anxiety disorders, personality disorders, and Alzheimer’s disease (7–11). However, these studies predominantly relied on qualitative indicators (e.g., whether the tree crown is closed). Similarly, scholars who applied the test in the diagnosis and treatment of schizophrenia also reported associations between certain qualitative indicators and the disorder (12). Nevertheless, most of the existing research has primarily focused on the relationship between qualitative indicators and schizophrenia, with relatively few studies attempting to integrate and standardize these indicators for quantitative analysis.

Currently, the application of the Tree Drawing Projection Test remains limited in several ways: reliance on qualitative indicators restricts its utility for rapid and large-scale screening and evaluation; and the manual measurement of certain quantitative features (e.g., area) is prone to error. To enhance its clinical applicability, our research team, building on previous studies examining the relationship between the Tree Drawing Projection Test and schizophrenia, integrated relevant indicators and developed specialized software by employing computer-based image recognition and data extraction techniques. Preliminary validation demonstrated the software’s effectiveness in distinguishing patients with schizophrenia from healthy controls (12). Our earlier findings also revealed that patients with schizophrenia tend to produce tree drawings with smaller crown and trunk indicators (e.g., area) compared to healthy controls.

The present study was therefore designed to further investigate the trajectory of changes in tree drawing indicators during treatment and rehabilitation among patients with schizophrenia, and to examine whether these indicators approach the levels observed in healthy controls as psychiatric symptoms improve. The ultimate aim was to provide objective and reliable evidence for evaluating treatment outcomes in schizophrenia.

## Methods

### Participant recruitment

The schizophrenia patient group in this study was recruited from the Department of Psychiatry at Liaocheng Veterans Hospital, Shandong Province, China, between October 2018 and November 2020.Inclusion criteria were as follows: (1) Diagnosis of schizophrenia in accordance with the Diagnostic and Statistical Manual of Mental Disorders, Fifth Edition (DSM-5) criteria for mental and behavioral disorders (13); (2) Age over 18 years, with no gender restrictions; (3) A total score > 35 on the Brief Psychiatric Rating Scale (BPRS) (4); (4) No prior formal training in drawing or painting.

The healthy control group was recruited from the same geographical region during the same period (2018–2020). Inclusion criteria for the control group were: (1) No psychotic symptoms as confirmed by a DSM-5-based diagnostic interview; (2) Absence of significant psychiatric symptoms, with no positive factors on the Symptom Checklist-90 (SCL-90), and no history of psychiatric disorders. The healthy control group was recruited to ensure comparability with the patient group in key demographic characteristics, particularly age and gender. Efforts were made to balance the two groups with respect to these factors in order to minimize potential confounding effects on study outcomes. A baseline comparison confirmed that there were no significant differences in age or gender distribution between the two groups (see **Table 1**).

**Table 1:** Gender and age distribution in the case and control groups (N [*%*] or Mean ± SD).

Variable	Case group	Control group	*t*	*p*
Gender			0.222	0.637
Male	32 (53.3)	34 (57.6)		
Female	28 (46.7)	25 (42.4)		
Age	44.98 ± 13.86	45.34 ± 11.47	-0.151	0.881

Exclusion criteria for both groups included: (1) Pregnancy, lactation, or menopausal status; (2) Substance abuse or dependence, or diagnosis of other severe psychiatric disorders; (3) Severe and unstable physical illnesses, including diagnosed diabetes, thyroid disorders, and hypertension.

### Ethical approval

This study was reviewed and approved by the Medical Ethics Committee of Liaocheng Veterans Hospital, Shandong Province (Approval No. 2021001). All participants and their legal guardians were fully informed of the study objectives and procedures prior to enrollment, and provided written informed consent to participate. Informed consent was obtained by the investigators of this study.

### Measures

#### Tree drawing projection test

Each participant was provided with a sheet of A4 paper and a black or blue-black gel pen. Drawing was conducted under standardized instructions, which included the following five main points:

1. The projection drawing test is not a test of drawing skills, and the quality of the drawing is not important.
2. The test is not a life drawing; the picture does not need to match real-world objects.
3. If the participant cannot draw what they want, they may draw a circle and write the Chinese characters for it.
4. Before drawing the tree, close your eyes and meditate for half a minute. Draw the tree that appears during meditation. If no tree appears, open your eyes and draw the tree you most want to draw.
5. After completing the drawing, write your age and gender on the paper (8).

#### High-resolution scanning

All tree drawings from the projective test were scanned using a Casio high-resolution scanner. The scanned images were saved in JPG format on a computer.

#### DSM-5

The Diagnostic and Statistical Manual of Mental Disorders (DSM), published by the American Psychiatric Association (APA), is the most widely used diagnostic manual for mental disorders in the United States and other countries. The first edition was published in 1952, and the fifth edition, DSM-5, was officially released on May 23, 2013 (13).

#### Tree drawing projection test analysis software

**Figure 1:**
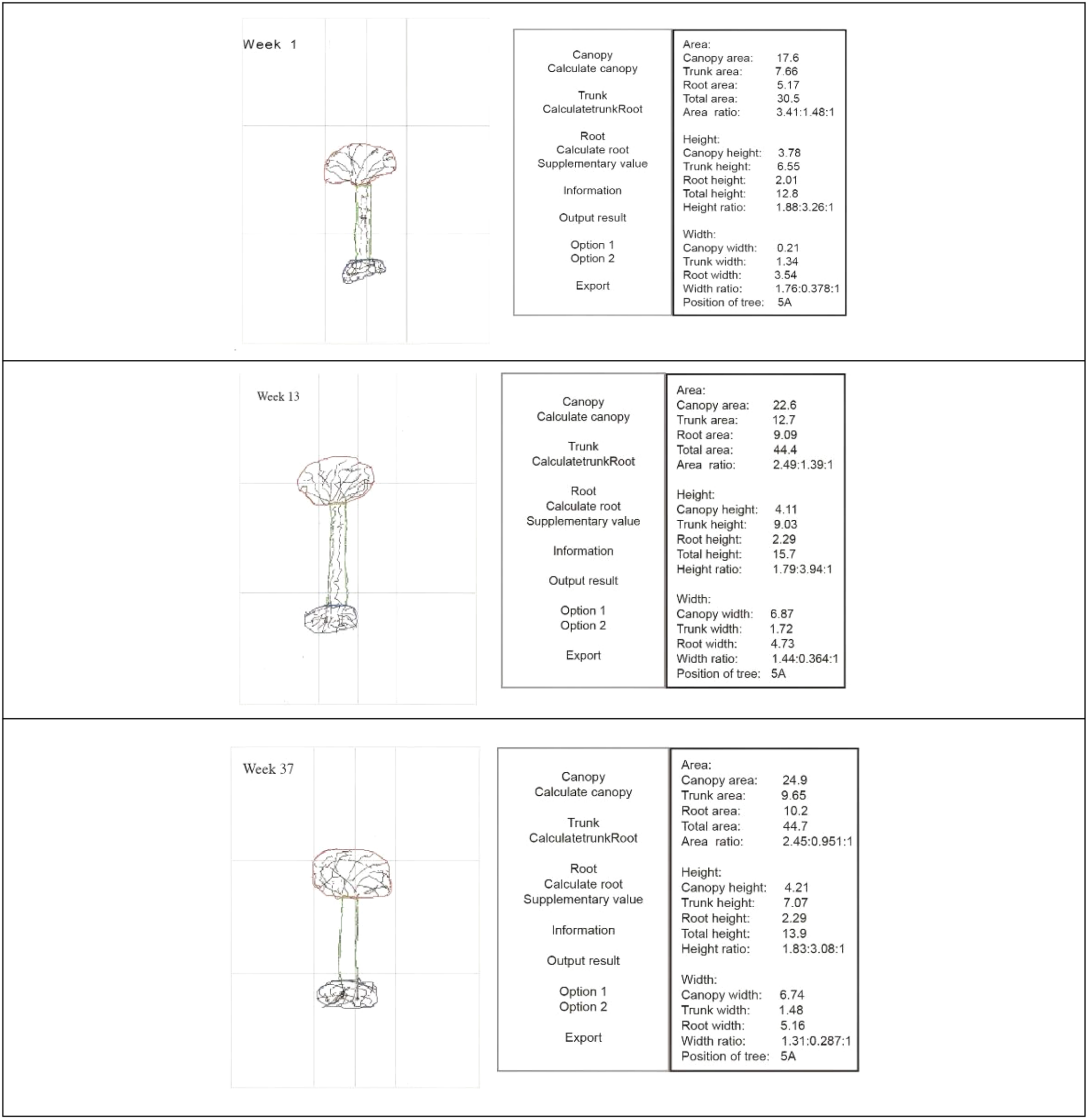
Tree drawing projection test: software scanning and data acquisition at different treatment time points.

The “Tree Drawing Projection Test Analysis Software”, developed by Professor Liu Wei from the Institute of Applied Psychology at Jiangsu University, was used to automatically calculate and extract data on the height, width, and area of the tree crown, trunk, and roots from the tree drawings. The data extracted by the software are shown in **Figure 1**, with measurement units in centimeters. This software received a National Intellectual Property Certificate in 2017 (14). The validity of the software has been verified in previous studies. Specifically, the software demonstrated the capacity to automatically and accurately compute quantitative parameters of the tree drawings, and clinical evaluations indicated that it could effectively differentiate patients with schizophrenia from healthy controls (12). When compared with manual measurements, the software exhibited a high level of accuracy in distinguishing between the two groups, thereby supporting its potential utility and reliability in clinical applications.

Regarding the algorithm, the software primarily employs an image recognition method in which the user traces the tree components (crown, trunk, and roots) directly on the digital image using a stylus or mouse. The software then automatically calculates and outputs quantitative parameters such as area, height, and width for each part. This semi-automated approach combines user-guided outlining with algorithmic computation to ensure both accuracy and efficiency in extracting measurements from complex drawings. Additionally, validation studies have demonstrated that the measurement error rate of the software is low (typically within ± 2–3% compared to manual measurements), confirming its high reliability and consistency in clinical and research applications.

### Study procedure

The treatment of schizophrenia is typically divided into three stages: the acute phase, consolidation phase, and maintenance phase. In this study, the phase definitions followed the authoritative Chinese textbook Psychiatry, which is widely used in clinical practice and psychiatric education in China. Specifically, the acute phase was defined as admission to week 12, with drawings collected within the first week of admission; the consolidation phase as week 13 to week 36, with drawings collected in the 13th week; and the maintenance phase as week 37 onwards, with drawings collected in the 37th week (15, 16). While there may be slight differences in time frames or terminology across international literature, these definitions are conceptually consistent with the internationally accepted terms “acute phase”, “stabilization/consolidation phase”, and “maintenance phase”.

All patients in the schizophrenia group received combined treatment with antipsychotic medication and psychotherapy during hospitalization. The pharmacological regimen primarily involved atypical antipsychotics, and benzodiazepines (e.g., diazepam) were not prescribed. Patients were discharged once psychiatric symptoms had remitted and the BPRS score had been reduced by ≥ 50%. Participant numbers were tracked throughout all treatment stages. No attrition occurred; however, withdrawal criteria were predefined, including voluntary discontinuation, clinical relapse, or rehospitalization after discharge.

### Data analysis

Data were organized and analyzed using SPSS version 26.0. Continuous variables were expressed as mean ± standard deviation (Mean ± SD). Independent-sample t-tests and analysis of variance (ANOVA) were employed as the primary statistical methods. Prior to analysis, all data were cleaned, including the removal of outliers and missing values. The normality of the data distribution was assessed using the Shapiro–Wilk test. For variables that did not meet the assumption of normality, appropriate transformations were applied, or nonparametric methods were used. Following ANOVA, Bonferroni correction was applied to adjust for multiple comparisons in order to control the family-wise error rate. A significance threshold of *p*<0.05 was adopted for all statistical tests.

*A priori* power analysis was conducted to determine the required sample size. Assuming a medium effect size (Cohen’s *d*=0.5), a significance level of 0.05, and a statistical power of 80%, the minimum required sample size for independent-sample t-tests was 64 participants per group (128 in total). For one-way ANOVA, assuming a small-to-medium effect size (*η²*=0.25), a significance level of 0.05, and a power of 80%, a minimum of 20 participants per group was required, yielding a total of 60 participants across three groups.

In this study, 60 patients with schizophrenia and 59 healthy controls were recruited. Although the sample size for the t-test comparisons was slightly below the *a priori* requirement, *post-hoc* power analysis based on Cohen’s *d*=0.377 for a representative comparison indicated that the actual statistical power was approximately 0.68, suggesting moderate power to detect medium effect sizes. For the ANOVA assessing BPRS scores across three time points, the estimated effect size was very large (*η²* ≈ 0.91, Cohen’s *f* ≈ 3.22), and *post-hoc* power analysis indicated an essentially 100% statistical power, demonstrating a very high ability to detect differences across the time points.

Overall, while slightly underpowered for some t-test comparisons, the study design provided adequate power for the main analyses and met methodological requirements for statistical validity.

## Results

### Sample

The case group comprised 60 patients with schizophrenia (age range: 21–83 years; mean ± SD: 44.98 ± 13.86), including 32 males and 28 females. The control group consisted of 59 individuals (age range: 20–80 years; mean ± SD: 45.34 ± 11.47), including 34 males and 25 females (**Table 1**). Baseline comparisons indicated no significant differences between the two groups in age (*t*=-0.151, *p*=0.881) or gender distribution (*χ^2^ =* 0.222, *p*=0.637). No participants withdrew during the study period.

### Temporal changes in tree drawing indicators: comparisons across three time points

**Figure 2:**
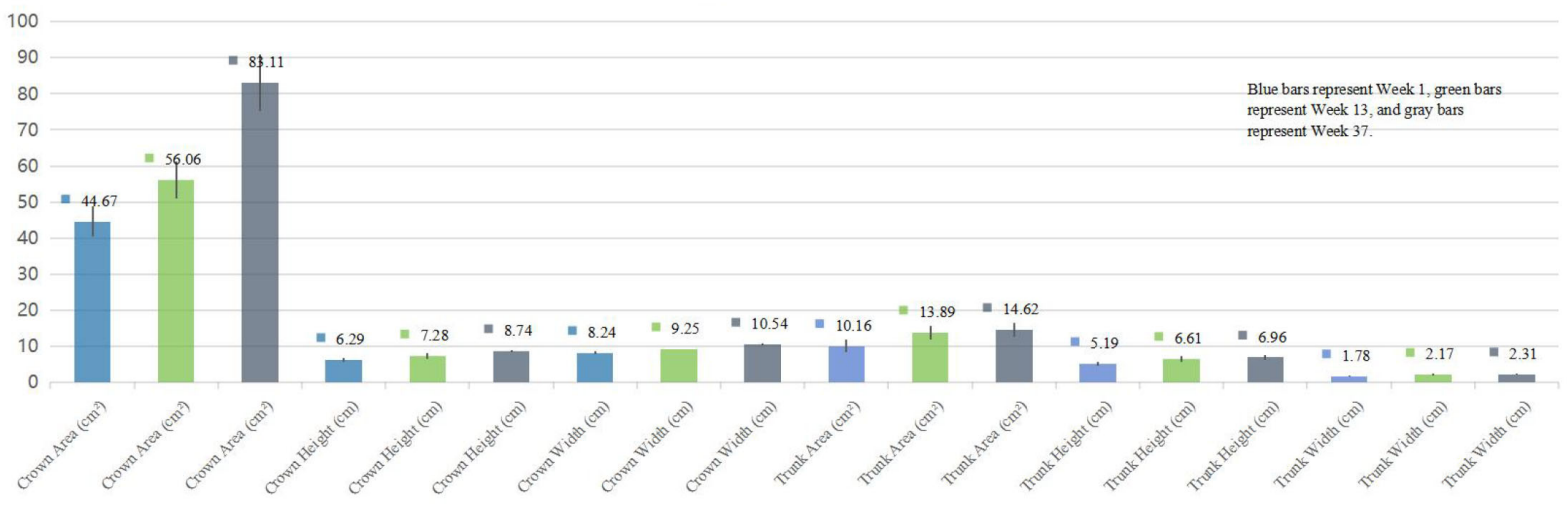
Comparative analysis of tree drawing parameters at different time points in patients with schizophrenia.

The distribution of tree-drawing indices met the assumption of normality (Shapiro–Wilk test). As shown in **Table 2**; **Figure 2**, patients with schizophrenia exhibited consistent increases in crown height, crown width, crown area, and trunk height from Week 1 to Week 13 and Week 37, and these differences were statistically significant (*p*<0.05). Trunk width, trunk area, root area, and root width also increased over time, although these changes were not statistically significant (*p* > 0.05).

**Table 2:** One-way ANOVA results comparing patient group across Week 1, Week 13, and Week 37 (n = 60).

Variable	Week 1 (Mean ± SD, 95% CI)	Week 13 (Mean ± SD, 95% CI)	Week 37 (Mean ± SD, 95% CI)	*F*	*p*	*η²*
Crown Area (cm²)	44.67 ± 5.31 (34.04, 55.30)	56.06 ± 7.25 (41.55, 70.55)	83.11 ± 12.63 (57.83, 108.38)	8.11	0.001	0.121
Crown Height (cm)	6.29 ± 0.65 (7.12, 9.36)	7.28 ± 0.76 (6.20, 8.35)	8.74 ± 0.25 (7.38, 10.10)	10.00	< 0.001	0.129
Crown Width (cm)	8.24 ± 0.56 (7.12, 9.36)	9.25 ± 0.53 (8.19, 10.31)	10.54 ± 0.56 (9.43, 11.65)	12.06	< 0.001	0.170
Trunk Area (cm²)	10.28 ± 1.67 (6.95, 13.62)	13.89 + 1.93 (10.02, 17.76)	14.62 + 1.79 (10.51, 18.74)	3.53	0.032	0.057
Trunk Height (cm)	5.19 ± 0.46 (4.33, 6.18)	6.61 ± 0.50 (5.61, 7.61)	6.96 ± 0.48 (5.99, 7.92)	6.89	0.001	0.106
Trunk Width (cm)	1.80 ± 0.19 (1.42, 2.20)	2.17 ± 0.25 (1.66, 2.68)	2.31 ± 0.25 (1.81, 2.81)	2.78	0.066	0.046
Root Area (cm²)	14.41 ± 1.20 (4.74, 27.21)	13.62 ± 1.53 (6.68, 28.11)	11.85 ± 1.29 (5.36, 22.91)	0.32	0.731	0.024
Root Height (cm)	2.75 ± 0.25 (1.77, 4.34)	2.56 ± 0.16 (2.12, 4.18)	2.34 ± 0.14 (1.81, 3.76)	0.55	0.827	0.041
Root Width (cm)	5.08 ± 0.77 (3.42, 6.75)	6.50 ± 1.29 (3.70, 9.29)	5.97 ± 0.98 (3.84, 8.09)	1.52	0.239	0.104
BPRS	53.40 ± 6.61 (51.69, 55.11)	23.07 ± 3.04 (22.28, 23.85)	22.60 ± 2.82 (21.87, 23.33)	920.31	< 0.001	0.912

Values are from one-way ANOVA comparing three time points (Week 1, Week 13, Week 37). *df*_1_ = 2, *df*_2_ = 177. Effect sizes are reported as partial eta squared (*η²*), calculated from the ANOVA model.

As shown in **Table 3**, *post hoc* analyses revealed significant changes in several tree drawing indices and BPRS scores over time. Although BPRS scores significantly decreased overall, the difference between week 13 and week 37 was not statistically significant (*p*=0.571). However, crown height, crown width, and crown area showed significant differences during the same interval (*p*<0.05).

**Table 3:** Pairwise comparisons of tree drawing indices and BPRS scores across treatment stages in patients with schizophrenia.

Variable	Week 1 vs week 13 (mean difference, 95% CI, *p*-value)	Week 13 vs week 37 (mean difference, 95% CI, *p*-value)	Week 1 vs week 37 (mean difference, 95% CI, *p*-value)	*η²*
Crown Area (cm²)	-11.38 (-19.89, -2.87), *p* =0.010	-27.06 (-62.27, -14.60), *p* =0.020	-38.44 (-19.89, -2.87), *p* =0.002	0.186
Crown Height (cm)	-0.99 (-1.80, -0.17), *p* =0.019	-1.46 (-2.67, -0.25), *p* =0.019	-2.45 (-3.67, -1.22), *p <* 0.001	0.225
Crown Width (cm)	-1.01 (-1.76, -0.26), *p* =0.010	-2.30 (-2.26, -0.32), *p <* 0.001	-2.30 (-3.37, -1.23), *p <* 0.001	0.244
Trunk Area (cm²)	-3.61 (-7.00, -0.22), *p* =0.037	-0.74 (-3.95, -2.48), *p* =0.648	-4.34 (-8.21, -0.47), *p* =0.028	0.093
Trunk Height (cm)	-1.36 (-2.27, -0.44), *p* =0.005	-0.35 (-1.29, 0.60), *p* =0.464	-1.70 (-2.75, -0.66), *p* =0.002	0.179
Trunk Width (cm)	-0.36 (-0.84, 0.12), *p* =0.138	-0.14 (-0.52, 0.24), *p* =0.455	-0.50 (-0.96, -0.05), *p* =0.031	0.078
Root Area (cm²)	-1.42 (-7.85, 5.01), *p* =0.641	3.26 (-6.41, 12.93), *p* =0.479	1.84 (-8.20, 11.81), *p* =0.699	0.045
Root Height (cm)	-0.10 (-0.85, 0.66), *p* =0.784	0.37 (-0.48, 1.22), *p* =0.359	0.28 (-0.50, 1.05), *p* =0.455	0.070
Root Width (cm)	-1.42 (-3.15, 0.32), *p* =0.101	0.53 (-1.39, 2.45), *p* =0.559	-0.88 (-2.55, 0.78), *p* =0.272	0.208
BPRS	30.33 (28.71, 31.96), *p* =0.001	0.47 (-1.16, 2.09), *p* =0.571	30.80 (29.18, 32.42), *p* =0.001	0.912

Mean difference = Score (Week 1) - Score (Week13), Score (Week 13) - Score (Week 37), Score (Week 1) - Score (Week 37). 95% CI represents the 95% confidence interval of the mean difference.

### Analysis of tree drawing indicator changes during the acute treatment phase by illness duration

As presented in **Table 4**, both patients with illness duration ≥ 10 years and those with duration < 10 years showed increases in crown area, crown height, and crown width from week 1 to week 13 (acute treatment phase); however, these changes were not statistically significant (*p* > 0.05). In contrast, trunk area, trunk height, and trunk width all decreased, and these differences were statistically significant (*p*<0.05).

**Table 4:** Changes in tree drawing indices during acute treatment in patients with different illness durations.

Variable	< 10 years (n = 38)	≥ 10 years (n = 22)	*t*	*p*
Crown Area (cm²)	42.38 ± 4.73	16.11 ± 1.41	0.785	0.436
Crown Height (cm)	2.49 ± 0.25	2.16 ± 0.34	0.194	0.847
Crown Width (cm)	2.63 ± 0.24	0.42 ± 0.03	1.492	0.141
Trunk Area (cm²)	5.66 ± 0.55	2.99 ± 0.21	2.701	0.012
Trunk Height (cm)	2.18 ± 0.20	0.94 ± 0.09	2.221	0.030
Trunk Width (cm)	0.68 ± 0.08	0.48 ± 0.05	3.653	0.001

Change = Index at Week 13 − Index at Week 1.

### Comparison of tree drawing indicators between case and control groups after the acute treatment phase

As shown in **Table 5**, at week 1, compared with the healthy control group, patients with schizophrenia exhibited significant differences in crown area, crown height, crown width, trunk area, trunk height, trunk width, root area, and root height (all *p*<0.05), whereas root width showed no significant difference. At week 13, significant group differences were observed in crown area, crown width, trunk area, trunk width, root area, and root height (all *p*<0.05); however, crown height, trunk height, and root width did not differ significantly. By week 37, significant differences remained in trunk width, root area, and root height (all *p*<0.05), while crown area, crown height, crown width, trunk area, trunk height, and root width were not significantly different between patients and controls.

**Table 5:** Comparison of tree drawing test measures between schizophrenia patients at three treatment time points (weeks 1, 13, and 37) and healthy control participants.

Variable	Week 1 (n = 60) vs control (n = 59)	Week 13 (n = 60) vs control (n = 59)	Week 37 (n = 60) vs control (n = 59)
Crown Area	-55.00 (-62.40, -47.60), *t* =-4.796, *p <*0.001, *d* =0.890	-43.61 (-50.30, -36.90), *t* =-3.211, *p* =0.002, *d*=0.595	-16.56 (-22.00, -11.10), *t* =-0.514, *p* =0.608, *d*=0.095
Crown Height	-2.95 (-4.31, -1.59), *t* =-3.499, *p* =0.001, *d*=0.638	-1.96 (-2.67, -1.25), *t* =-2.023, *p* =0.450,*d*=0.374	-0.50 (-1.68, 0.68), *t* =0.014, *p* =0.989, *d*=0.003
Crown Width	-3.75 (-5.11, -2.39), *t* =-4.618, *p <*0.001, *d* =0.854	-2.74 (-3.40, -2.08), *t* =-5.875, *p <*0.001, *d*=1.085	-1.45 (-3.06, 0.16), *t* =-1.875, *p* =0.063, *d*=0.347
Trunk Area	-13.78 (-16.25, -11.31), *t* =-3.579, *p* =0.001, *d*=0.661	-10.05 (-12.31, -7.79), *t* =-1.974, *p* =0.049, *d*=0.367	-9.32 (-11.9, -6.74), *t* =-1.657, *p* =0.100, *d*=0.308
Trunk Height	-1.69 (-2.25, -1.13), *t* =-2.057, *p* =0.042, *d*=0.380	-0.27 (-0.87, 0.33), *t* =-0.280, *p* =0.780, *d*=0.052	0.08 (-0.33, 0.49), *t* =0.845, *p* =0.400, *d*=0.006
Trunk Width	-2.55 (-3.04, -2.06), *t* =-5.521, *p <*0.001, *d*=1.021	-2.16 (-2.78, -1.54), *t* =-3.993, *p <*0.001, *d*=0.742	-2.02 (-2.52, -1.52), *t* =-3.670, *p <*0.001, *d*=0.682
Root Area	7.98 (7.25, 8.71), *t* =2.690, *p* =0.009, *d* =0.601	7.19 (6.46, 7.92), *t* =2.862, *p* =0.005, *d*=0.636	42.00 (4.77, 6.07), *t* =2.582, *p* =0.012, *d* =0.560
Root Height	1.99 (1.90, 2.08), *t* =6.152, *p <*0.001, *d* =1.374	1.80 (1.72, 1.88), *t* =6.713, *p <*0.001, *d* =1.490	1.58 (1.50, 1.66), *t* =6.678, *p <*0.001, *d* =1.450
Root Width	3.00 (2.64, 3.36), *t* =0.329, *p* =0.744, *d* =0.094	3.77 (3.39, 4.15), *t* =0.849, *p* =0.400, *d* =0.240	3.57 (3.21, 3.93), *t* =0.789, *p* =0.434, *d* =0.215

Control Group (Mean ± SD): Crown Area (cm²) = 99.67 ± 10.89, Crown Height (cm) = 9.24 ± 2.84, Crown Width (cm) = 11.99 ± 2.15, Trunk Area (cm²) = 23.94 ± 4.23, Trunk Height (cm) = 6.88 ± 1.53, Trunk Width (cm) = 4.33 ± 0.92, Root Area (cm²) = 6.43 ± 1.61, Root Height (cm) = 0.76 ± 0.07, Root Width (cm) = 2.13 ± 0.21. *Δ* represents the mean difference between the experimental group and the control group. Welch’s independent-samples t-test was used to account for unequal variances; degrees of freedom (*df*) were calculated using the Welch–Satterthwaite formula. Cohen’s *d* was calculated using the pooled standard deviation. 95% CI represents the 95% confidence interval of the mean difference. n indicates the sample size of each group.

## Discussion

According to the theoretical assumptions of the Tree Drawing Projection Test, the tree crown is hypothesized to reflect an individual’s psychological domain (11, 17). The present study found that both the trees and tree crowns drawn by individuals with schizophrenia were smaller in area compared to those of the normal control group, accurately reflecting underlying mental issues. These may include positive symptoms such as thought insertion, persecutory delusions, and hallucinations, which contribute to patients’ reluctance to engage with others and venture outside. In daily life, these issues manifest as weakness, social withdrawal, poor interpersonal skills, and low social adaptability (10). Previous studies have similarly indicated that a smaller tree crown area in the Tree Drawing Test reflects low internal psychological drive, feelings of inferiority, lack of vitality, poor self-control, dependency, withdrawal, detachment from reality, and personality disintegration in patients with schizophrenia. In this study, the crown area, crown height, and crown width of patients in the schizophrenia group showed steady increases during treatment and progressively approached the levels observed in the control group, indicating an alleviation of thought-related symptoms and a therapeutic effect.

In comparing disease course subgroups, patients with a course of schizophrenia shorter than 10 years showed some increase in crown area, height, and width after the acute treatment phase (week 13), compared to baseline. However, the differences were not statistically significant when compared to the group with a disease duration over 10 years. It is known that cognitive impairments are already present before or at the onset of schizophrenia, with at least 85% of patients experiencing persistent and severe cognitive deficits (18), particularly in areas such as attention, verbal memory, and executive functioning. Some studies suggest that these cognitive impairments remain relatively stable over time, with little variation in severity across different disease durations (19), which may explain the current findings. Additionally, the fact that tree crown area and width in patients after acute treatment did not reach the levels of the normal control group supports this viewpoint. Some research indicates that the first five years following the onset of schizophrenia represent a critical treatment window. During this period, neuroinflammation and other pathological processes can cause significant neural damage. In particular, the volume of grey matter in the parietal and frontal lobes of first-episode schizophrenia patients tends to decrease most rapidly within the first 3–5 years, with minimal changes thereafter (20). In this study, there were no participants with a disease duration under five years. Future studies should consider including patients with disease duration shorter than five years or those experiencing their first episode of schizophrenia for comparative analysis.

In this framework, the tree trunk is commonly interpreted as an indicator of an individual’s emotional state (9, 21). Patients with schizophrenia often exhibit emotional disturbances, mainly characterized by blunted affect, diminished or indifferent emotional responses to external stimuli, emotional incongruity, or even emotional paradoxes. Most patients are unaware of their loss of emotional expressiveness (22). Previous studies have shown that the trunk area and trunk width of tree drawings by patients with schizophrenia are significantly smaller than those of healthy controls, reflecting emotional blunting, shallow affect, and emotional dissonance (23). The present study also found a statistically significant increase (*p*<0.05) in trunk height, trunk width, and trunk area across the acute treatment phase, consolidation phase, and maintenance phase. These findings suggest that antipsychotic treatment has a positive effect on emotional functioning in schizophrenia. However, although trunk area and width increased after the acute treatment phase, they still did not reach the levels observed in the normal control group. This indicates that while emotional symptoms improve with medication, most patients with schizophrenia continue to experience emotional blunting and deterioration, preventing a full return to normal emotional functioning (24, 25). A subgroup analysis based on illness duration showed that, for both patients with illness duration less than 10 years and those with more than 10 years, trunk area, height, and width changed significantly before and after the acute treatment phase. This suggests that emotional functioning is more likely to recover in patients with a shorter illness duration, whereas recovery is limited in those with a longer disease course (26).

The tree roots, in turn, are often hypothesized to represent psychological constructs associated with an individual’s sense of security, fundamental drives, or latent impulses (27). Schizophrenia is primarily characterized by disturbances in thought and emotional functioning, often accompanied by cortical disinhibition and heightened instinctual drives (7). In this study, the schizophrenia group exhibited greater root area, height, and width than the control group at Week 13, with statistically significant differences. Over the course of treatment, root area gradually decreased, paralleling the alleviation of psychotic symptoms, although values remained higher than those of the control group. These changes align with the direction of clinical symptom improvement—i.e., as symptoms abate, root-related metrics tend to decline—suggesting that quantitative root indicators may serve as projective measures reflecting changes in patients’ clinical states. These findings are consistent with previous studies indicating that projective tests can capture variations in clinical status.

Some researchers have linked certain clinical manifestations of schizophrenia, such as impulsivity or emotional/behavioral dysregulation, to reduced cortical control or imbalances in cortico-cortical and cortico-limbic circuits (28). However, directly mapping a single drawing feature onto a specific neurobiological process (e.g., “cortical disinhibition leading to heightened instinct”) represents a cross-level inference and entails a degree of extrapolation. Therefore, in this study, potential biological explanations are considered within a hypothetical framework: increases in root indicators may co-occur with neural dysfunctions related to emotional or drive representations, but they should not be interpreted as direct biological surrogates or causal evidence.

Future studies could adopt multimodal designs, integrating tree drawing metrics with standardized clinical scales, systematic neuropsychological assessments, and neuroimaging measures, while accounting for potential confounders such as medication type/dose, illness duration, and comorbidities. Only through converging evidence from multiple sources can the relationship between projective drawing features and specific biological processes be more reliably delineated.

This study found that, compared with the BPRS, the Tree Drawing Projection Test showed statistically significant changes in crown area, height, and width at weeks 13 and 37, whereas the BPRS scores, although altered, did not reach statistical significance. Previous research has indicated that scale scores may only change when schizophrenia symptoms reach a certain threshold, and the assessment process of these scales still involves some subjective components (29). The BPRS is relatively insensitive to negative symptoms characterized primarily by affective blunting and social withdrawal, particularly during the maintenance phase of schizophrenia, when the focus is on the restoration of social functioning. This may explain why BPRS scores exhibited changes at weeks 13 and 37 without achieving statistical significance.

## Conclusion

In summary, this study demonstrates that during the course of schizophrenia treatment, certain indices of the Tree Drawing Projection Test change in correspondence with symptom improvement over time. Notably, crown-related indices exhibited larger effect sizes (*η²*) than trunk-related indices, suggesting that the Tree Drawing Projection Test can serve as an auxiliary tool for evaluating treatment efficacy and monitoring symptom progression. In particular, during the maintenance phase of schizophrenia, the Tree Drawing Projection Test may provide valuable information for assessing subtle changes in clinical status.

Despite these findings, several limitations exist. First, it was conducted at a single center with a relatively small sample size, which may limit the representativeness of the findings and the statistical power. Future studies should consider expanding the sample size to enhance the stability and generalizability of the results. Second, this study did not include patients with illness duration less than five years, particularly those with first-episode or early-stage schizophrenia, limiting comprehensive understanding of psychological changes in the early course of the disorder. Furthermore, medication types and dosages, psychosocial interventions, and comorbidities were not controlled, which may have confounded the tree drawing indicators and influenced result interpretation.

Future research should consider integrating the Tree Drawing Projection Test with standardized clinical assessments, neuropsychological testing, and neuroimaging modalities to improve its reliability and validity and further clarify its auxiliary role in treatment evaluation for schizophrenia. Additionally, improvements could include larger samples, inclusion of first-episode patients, diversification of assessment methods, and adoption of longitudinal designs to dynamically track changes in tree drawing indicators throughout treatment. These steps aim to provide a more comprehensive evaluation of schizophrenia treatment outcomes and offer stronger scientific evidence for clinical practice.

## Data Availability

The raw data supporting the conclusions of this article will be made available by the authors, without undue reservation.
